# Induction of Heme Oxygenase-1 Inhibits Cell Death in Crotonaldehyde-Stimulated HepG2 Cells via the PKC-δ -p38 -Nrf2 Pathway

**DOI:** 10.1371/journal.pone.0041676

**Published:** 2012-07-25

**Authors:** Seung Eun Lee, Hana Yang, Seong Il Jeong, Young-Ho Jin, Cheung-Seog Park, Yong Seek Park

**Affiliations:** 1 Department of Microbiology, School of Medicine, Kyung Hee University, Seoul, Republic of Korea; 2 Department of Physiology, School of Medicine, Kyung Hee University, Seoul, Republic of Korea; Children's Hospital Boston & Harvard Medical School, United States of America

## Abstract

**Background:**

Crotonaldehyde, an alpha, beta-unsaturated aldehyde present in cigarette smoke, is an environmental pollutant and a product of lipid peroxidation. It also produces adverse effects to humans and is considered as a risk factor for various diseases. Heme oxygenase-1 (HO-1) plays important roles in protecting cells against oxidative stress as a prime cellular defense mechanism. However, HO-1 may be associated with cell proliferation and resistance to apoptosis in cancer cells. The aim of this study was to examine the effects of HO-1 induction on cell survival in crotonaldehyde-stimulated human hepatocellular carcinoma (HepG2) cells.

**Methods:**

To investigate the signaling pathway involved in crotonaldehyde-induced HO-1 expression, we compared levels of inhibition efficiency of specific inhibitors and specific small interfering RNAs (siRNAs) of several kinases. The cell-cycle and cell death was measured by FACS and terminal dUTP nick-end labeling (TUNEL) staining.

**Results:**

Treatment with crotonaldehyde caused a significant increase in nuclear translocation of NF-E2 related factor (Nrf2). Treatment with inhibitors of the protein kinase C-δ (PKC-δ) and p38 pathways resulted in obvious blockage of crotonaldehyde-induced HO-1 expression. Furthermore, treatment with HO-1 siRNA and the specific HO-1 inhibitor zinc-protoporphyrin produced an increase in the G_0_/G_1_ phase of the cell cycle in crotonaldehyde-stimulated HepG2 cells.

**Conclusions:**

Taken together, the results support an anti-apoptotic role for HO-1 in crotonaldehyde-stimulated human hepatocellular carcinoma cells and provide a mechanism by which induction of HO-1 expression via PKC-δ–p38 MAPK–Nrf2 pathway may promote tumor resistance to oxidative stress.

## Introduction

Crotonaldehyde is a highly reactive alpha, beta-unsaturated aldehyde that occurs naturally in many foods and which is also generated from lipid peroxidation [1], [2]. Cigarette smoke is generally considered to be the main source of human exposure to crotonaldehyde [3]. High levels of crotonaldehyde have been measured in cigarette smoke (1.4–15 µg/cigarette) [4], a level that is approximately 2,300-fold higher than benzo[a]pyrene, an extremely diffuse environmental pollutant [5].

Crotonaldehyde produces adverse effects in humans. It is highly toxic in the eye, respiratory tract, and to the skin [6], [7], and is also mutagenic in numerous cell systems and induces liver tumors in rodents [8], [9], [10]. Crotonaldehyde is considered as a risk factor for various diseases such as neurodegenerative disorders [1] and chronic pulmonary disease [11]. The toxicity of crotonaldehyde is a consequence of its strongly reactive, electrophilic carbonyl group, which can rapidly react with cellular nucleophiles, causing extensive protein and DNA modifications and obstructing normal cellular function [12], [13], [14]. Several studies have revealed that crotonaldehyde reduces glutathione (GSH) levels and enhances the levels of intracellular reactive oxygen species (ROS), leading to a state of vulnerability in human airway cells [2], [11]. Crotonaldehyde has been reported to modulate biological reactions by a variety of downstream signaling pathways [15], [16].

Heme oxygenase-1 (HO-1) is one of the cytoprotective enzymes in response to a variety of stimuli, including cellular oxidative stress. HO-1 controls the initial and rate-limiting steps in heme catabolism, catalyzing the conversion of heme into biliverdin, free iron, and carbon monoxide [17]. HO-1 has been shown to play a central role in cytoprotective mechanisms, such as anti-inflammatory [18], anti-proliferative [19], and anti-apoptotic properties [20]. In addition, HO-1 exerts positive effects on angiogenesis [21] and on proliferation of sarcoma and hepatoma cells *in vivo* [22]. On the other hand, the cytoprotective effect of HO-1 might modify the endogenous balance between apoptosis and proliferation towards an anti-apoptotic and pro-proliferative status, which are relevant to oncogenesis, maintenance, and resistance to chemotherapy.

Thus, we hypothesized that crotonaldehyde induces HO-1 expression via Nrf2 pathways in hepatocellular carcinoma and that induction of HO-1 expression will have an anti-apoptotic effect. The results of the present study support a potential function for HO-1 induction in providing a survival advantage for carcinoma cells in conditions of exposure to environmental pollutants, including crotonaldehyde.

## Results

### Treatment with Crotonaldehyde Induces HO-1 Expression in HepG2 Cells

**Figure 1:**
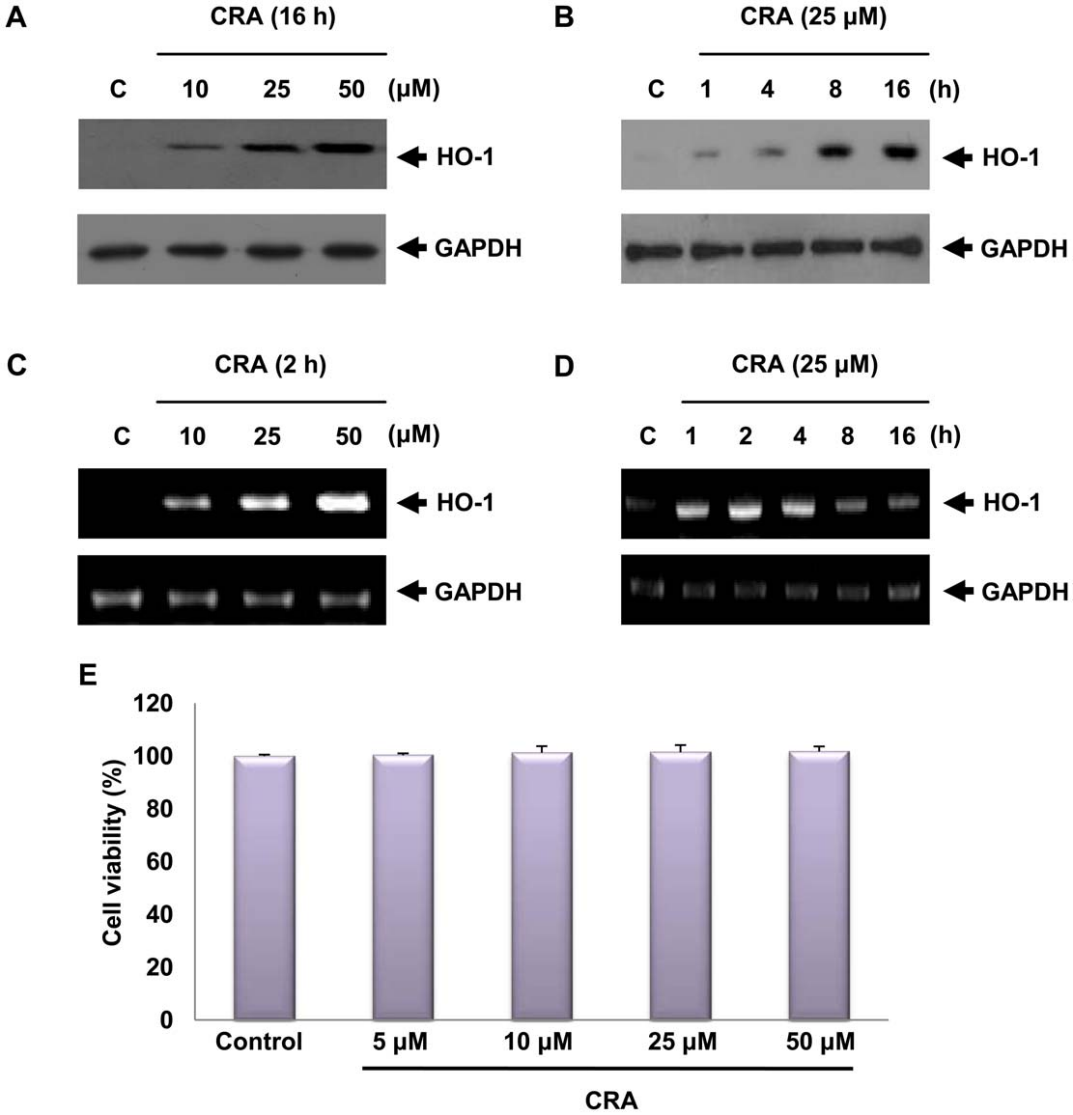
Effect of crotonaldehyde (CRA) on HO-1 expression. Cells were incubated with the indicated concentration of crotonaldehyde for 16 h. Protein in cell lysates was analyzed by Western blot using HO-1 specific antibody (A). RT-PCR was conducted to measure the levels of HO-1 mRNA transcript (C). Cells were harvested at various time intervals (B and D). Glyceraldehyde 3-phosphate dehydrogenase (GAPDH) levels were measured to ensure equal amounts of protein and mRNA loaded. Cell viability was estimated by the MTT method (E). Data represent the mean ± SD of results in three independent experiments.

HO-1 is an important stress-responsive antioxidant enzyme that is induced by a variety of oxidative stimuli. Treatment of HepG2 cells with crotonaldehyde produced a significant increase in the expression of HO-1 protein and mRNA in a dose-dependent manner (Figure 1A and 1C). Upon exposure to 25 µM crotonaldehyde, HO-1 mRNA expression increased rapidly and preceded HO-1 protein accumulation (Figure 1B and 1D). Cell viability was evaluated by the MTT-based assay after 16 h of stimulation with various concentrations of crotonaldehyde (Figure 1E). At the concentrations used in this experiment, crotonaldehyde did not influence cell viability. These results suggest that concentrations of crotonaldehyde below 50 µM are not toxic to HepG2 cells.

**Figure 2:**
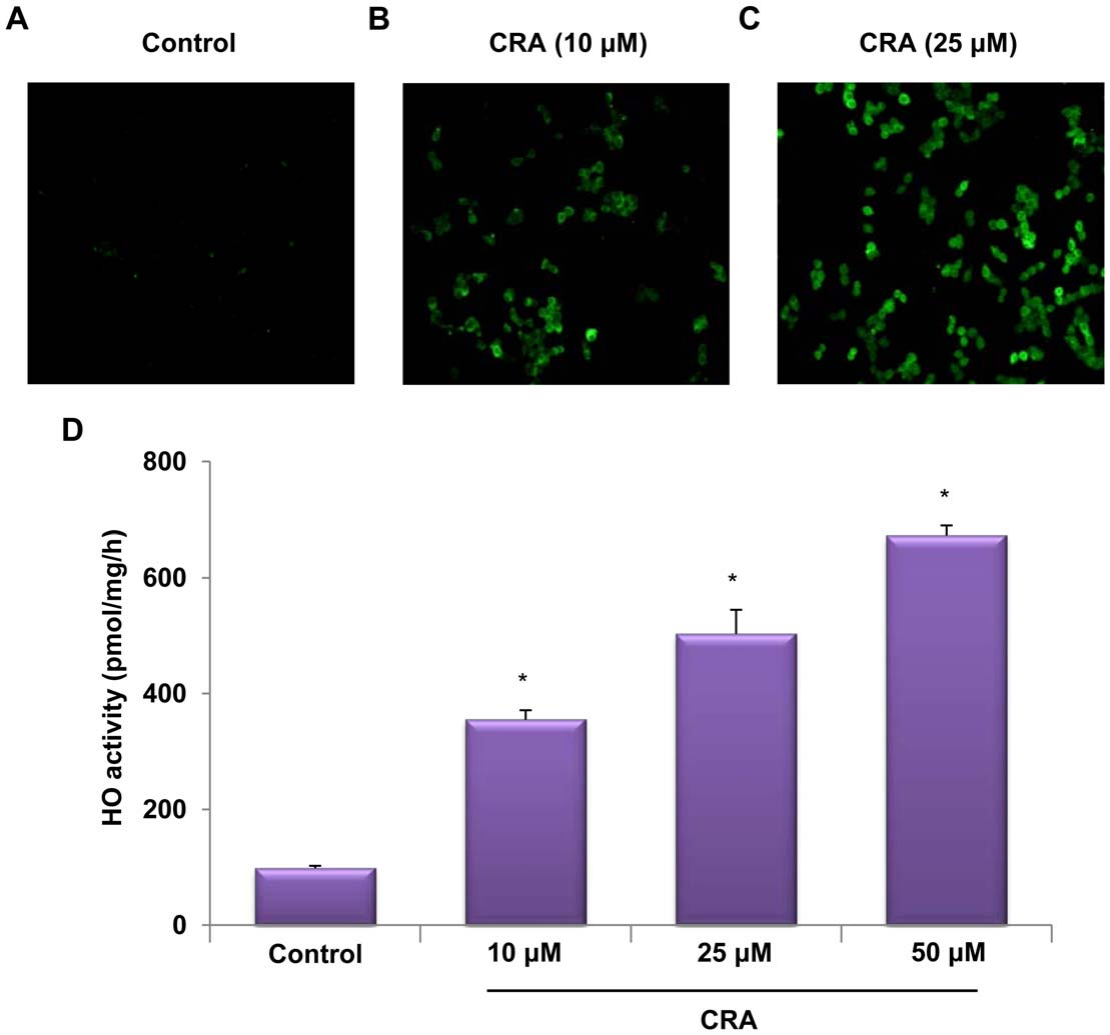
Induction of HO-1 expression in crotonaldehyde-stimulated HepG2 cells. Immunofluorescence staining for HO-1 revealed increased expression of HO-1 in cells treated for 16 h with 10 and 25 µM crotonaldehyde, compared with non-treated cells (A, B, and C). Cells were fixed and HO-1 localization was determined by immunofluorescence staining with an anti-HO-1 antibody followed by a fluorescence-tagged secondary antibody. HO activity was measured in cells at 16 h after treatment of cells with 10, 25, and 50 µM crotonaldehyde (C). Each bar represents the mean ± SD of three independent experiments. *p<0.05 vs. control.

After treatment with 10 and 25 µM crotonaldehyde, HO-1 localization was determined in fixed cells by immunofluorescence staining. Immunofluorescence analysis revealed that HO-1 protein levels were markedly increased in HepG2 cells treated with crotonaldehyde (Figure 2A, control vs. Figure 2B and 2C, crotonaldehyde-treated cells). To confirm the effects of crotonaldehyde on HO-1 induction, HO-1 activity was determined in crotonaldehyde-stimulated HepG2 cells. Exposure of cells to various concentrations of crotonaldehyde for 16 h resulted in obviously increased HO-1 activity compared to the untreated cells (Figure 2D) (untreated cells, *p<0.05). These data confirmed that up-regulation of HO-1 mRNA and protein was accompanied by increased HO-1 activity in crotonaldehyde-stimulated cells.

### HO-1 Up-regulation Induced by Crotonaldehyde is Mediated by the Protein Kinase C (PKC)-δ and p38 Mitogen-activated Protein Kinase (MAPK) Pathways

**Figure 3:**
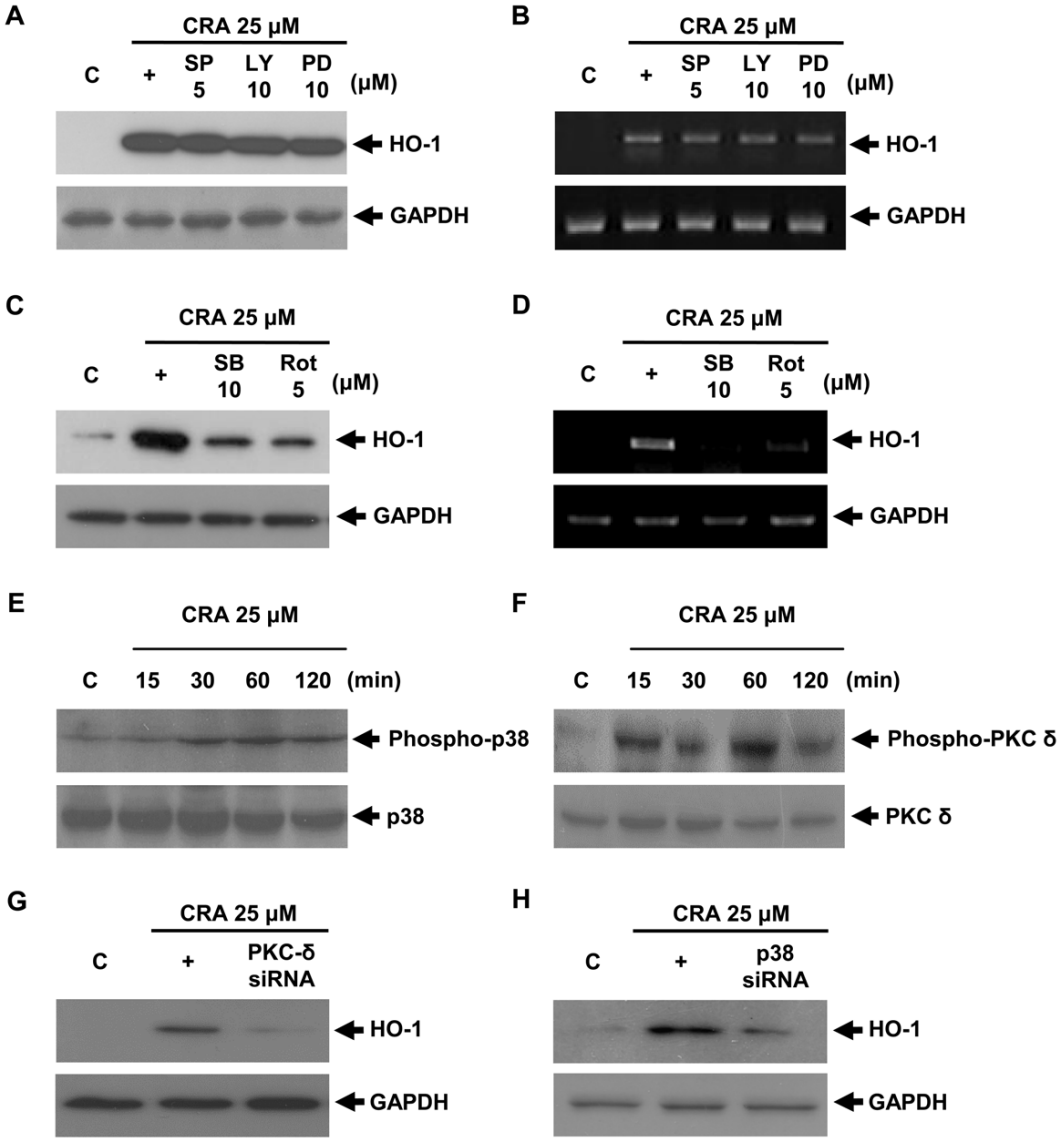
Involvement of PKC-δ and p38 signaling pathways in crotonaldehyde-stimulated HO-1 expression. Cells were pretreated with PD 98059 (MEK1 inhibitor), LY 294002 (Akt/PI3K inhibitor), SP 600125 (c-Jun N-terminal Kinases (JNKs) inhibitor), SB 203580 (p38 inhibitor) or Rottlerin (PKC-δ inhibitor) for 1 h, followed by incubation with 25 µM crotonaldehyde for 16 h. Whole cell lysates were prepared and subjected to western blot analysis with antibodies against anti-HO-1 and GAPDH, as indicated (A and C). HepG2 cells were pretreated with inhibitors at the indicated concentrations for 1 h; followed by incubation with 25 µM crotonaldehyde for 2 h. Total RNA was prepared and subjected to RT-PCR for HO-1 and GAPDH (B and D). Cell lysates were immunoblotted with antibodies for the phosphorylated form of p38 MAPK and PKC-δ (E and F). Transient transfection of cells with PKC-δ or p38 siRNA inhibited up-regulation of crotonaldehyde-stimulated HO-1 protein expression (G and H). Representative Western blots of three independent experiments are shown. +, crotonaldehyde alone treated group.

Since several signaling pathways are involved in induction of HO-1 expression [23], [24]. It was of interest to assess the involvement of the signaling pathway in the observed effects of crotonaldehyde. To investigate the upstream signaling pathway involved in crotonaldehyde-induced HO-1 expression, we next compared levels of inhibition efficiency of specific inhibitors of the PKC, MARK, and phosphoinositol-3-kinase (PI3K) pathways. Specifically, inhibitors of the PKC-δ and p38 pathways were strongly reduced HO-1 induction in crotonaldehyde-stimulated HepG2 cells (Figure 3A, 3B, 3C and 3D). We measured the activation of PKC-δ and p38 by detection of increased phospho-PKC-δ and p38 levels in crotonaldehyde-stimulated HepG2 cells and found that phosphorylation of PKC-δ and p38 increased in crotonaldehyde-stimulated HepG2 cells (Figure 3E and 3F). In a time-course study, crotonaldehyde led to an early peak (≈ 1 h) of phospho-PKC-δ and p38 induction. We therefore confirmed the involvement of PKC-δ and p38 signaling in crotonaldehyde-induced HO-1 expression by using specific small interfering RNAs (siRNAs) for PKC-δ and p38. Transient transfection with PKC-δ or p38 siRNA decreased PKC-δ or p38 protein expression (data not shown), and PKC-δ and p38 siRNA abolished crotonaldehyde-mediated induction of HO-1 protein (Figure 3G and 3H), indicating the involvement of the PKC-δ and p38 signaling pathways in crotonaldehyde-stimulated HO-1 induction.

### Crotonaldehyde Enhances HO-1 Induction Responsible for Nuclear Translocation of NF-E2 Related Factor (Nrf2)

**Figure 4:**
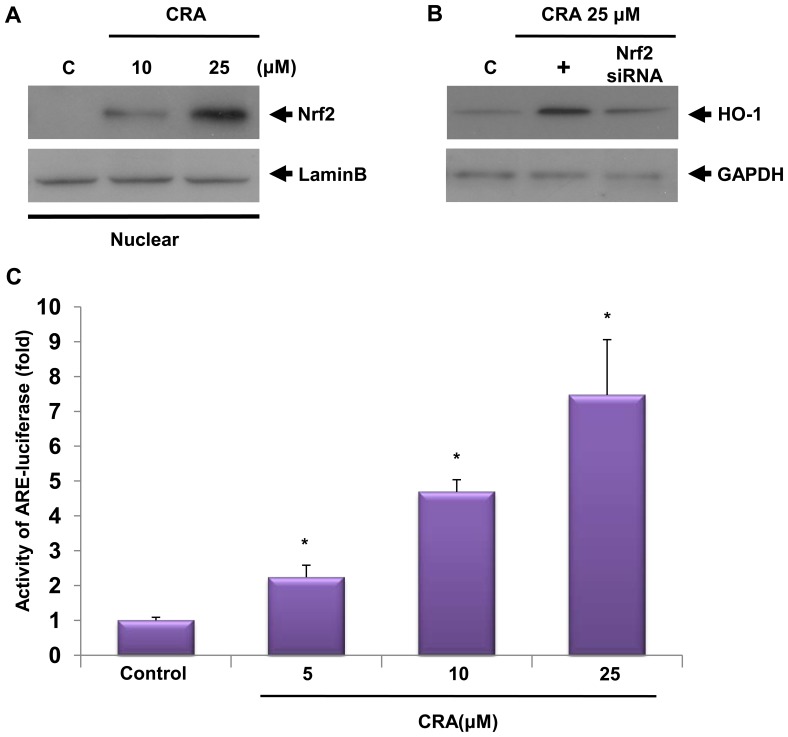
Crotonaldehyde-stimulated induction of HO-1 expression is mediated by Nrf2-EpRE/ARE. Cells were treated with crotonaldehyde at the indicated concentrations for 4 h. Nuclear extracts were prepared, and protein samples (40 µg) were subjected to Western blotting using an anti-Nrf2 antibody or an anti-Lamin B (a nuclear protein marker) antibody (A). Transient transfection of HepG2 cells with Nrf2 specific siRNA inhibited expression of the HO-1 protein. Nrf2 siRNA abrogated induction of crotonaldehyde-stimulated HO-1 protein expression (B). +, crotonaldehyde alone treated group. Cells transfected with an EpRE/ARE-luciferase construct were treated with various concentrations of crotonaldehyde for 4 h, and the lysates were mixed with a luciferase substrate. A luminometer was used to measure luciferase activity (C). Data represent the mean ± SD of 4 independent experiments. *p<0.001 vs. control.

Nrf2 is a redox-sensitive basic-leucine zipper transcription factor. The induction of phase II detoxifying and antioxidant enzymes, including HO-1, is regulated by the Nrf2/Keap1 transcription factor system [25]. The primary control of Nrf2 transcriptional activation of phase II gene induction relies on subcellular distribution in response to oxidative or electrophilic stress. Thus, we determined nuclear levels of Nrf2 in control and crotonaldehyde-stimulated cells to investigate whether the induction of HO-1 expression in crotonaldehyde-stimulated cells is mediated by Nrf2. To assess whether crotonaldehyde stimulates Nrf2 translocation in HepG2 cells, cells were treated with various concentrations of crotonaldehyde for 4 h, and the nuclear fractions were extracted for preparation of nuclear proteins. Nrf2 proteins in the cellular nuclear compartments were detected by Western blot. As shown in Figure 4A, Nrf2 was translocated to nuclei following treatment of cells with crotonaldehyde in a dose-dependent fashion. We also assessed the effect of transient transfection with Nrf2 siRNA on crotonaldehyde-stimulated HO-1 expression. Silencing with Nrf2 siRNA reduced Nrf2 proteins compared to negative controls in total cell lysates (data not shown). The crotonaldehyde-induced increase in HO-1 protein was eliminated by transfection with Nrf2 siRNA (Figure 4B), suggesting that crotonaldehyde-stimulated induction of HO-1 expression requires activation of Nrf2.

The promoter region of the human HO-1 gene harbors consensus electrophile/antioxidant response element (EpRE/ARE) sequences that are essential for the up-regulated expression of phase II detoxifying and antioxidant enzymes in response to oxidative stress, and Nrf2 activates transcription of its target genes through binding specifically to EpRE/ARE found in the gene promoters [25], [26]. To examine the role of Nrf2 in inducing the EpRE/ARE-dependent transcriptional activity, cells were transiently transfected with an EpRE/ARE luciferase reporter plasmid, treated with various concentrations of crotonaldehyde for 4 h, and luciferase activity was determined. In agreement with the data concerning nuclear localization, exposure of cells transfected with an EpRE/ARE-Luc vector to crotonaldehyde resulted in a marked up-regulation of EpRE/ARE-luciferase activity (Figure 4C, *p*<0.01), indicating that Nrf2- EpRE/ARE is an important transcription factor responsible for crotonaldehyde-stimulated HO-1 induction.

### Effect on Cell Cycle Distribution After Exposure to Zinc-protoporphyrin (ZnPP) or HO-1 siRNA in Crotonaldehyde-stimulated HepG2 Cells

**Figure 5:**
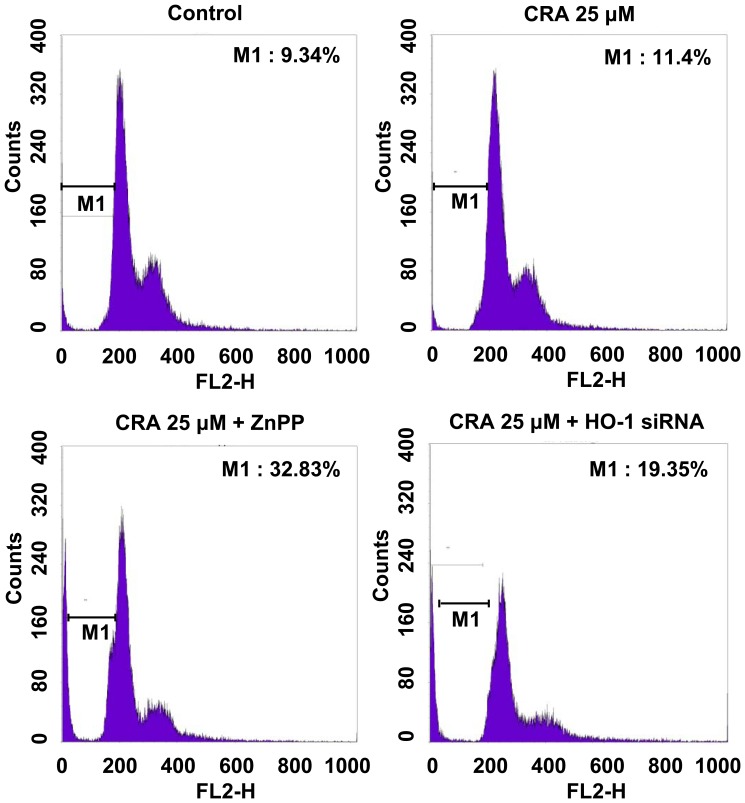
Effect on cell cycle distribution after treatment with ZnPP or HO-1 siRNA in crotonaldehyde-stimulated HepG2 cells. Cell cycle analysis was performed by PI staining. HepG2 cells were treated with 25 µM crotonaldehyde and absence or presence of 1 µM ZnPP or HO-1 siRNA for 16 h. After treatment, the cells were stained with propidium iodide. Fluorescence activated cell sorting (FACS) analysis using PI staining was performed for DNA content measurement. Apoptosis was measured as the percentage of total cell population with the sub-G1 DNA content and was in the region labeled M1. Results are expressed as a dot plot and represent three independent experiments.

HO-1 is involved in regulation of cell cycle, although these effects are tissue-dependent. In vascular endothelium, induction of HO-1 has been shown to stimulate cell cycle progression, whereas in vascular smooth muscle cells it can attenuate proliferation [27], [28]. To further determine whether the increased level of HO-1 activity enhanced by crotonaldehyde confers cytoprotection against oxidative stress, HepG2 cells were pretreated with a specific HO-1 inhibitor, ZnPP, and HO-1 siRNA. Crotonaldehyde-stimulated cells were pre-incubated with or without 1 µM ZnPP or HO-1 siRNA, and cell cycle distribution was detected by fluorescence-activated cell sorting (FACS) analysis. PI staining followed by FACS analysis showed that, while there was a minimal increase in the sub-G1 phase, indicative of apoptosis, in crotonaldehyde-stimulated cells, HO-1 siRNA or ZnPP-pretreated cells displayed marked accumulation of cells in the sub-G1 phase following treatment with crotonaldehyde, compared to untreated cells (Figure 5). Thus, these results suggest that induction of HO-1 expression by crotonaldehyde is affecting cell resistance to apoptosis.

### Pharmacological Inhibition of HO-1 Increases Cell Death in Crotonaldehyde-stimulated HepG2 Cells

**Figure 6:**
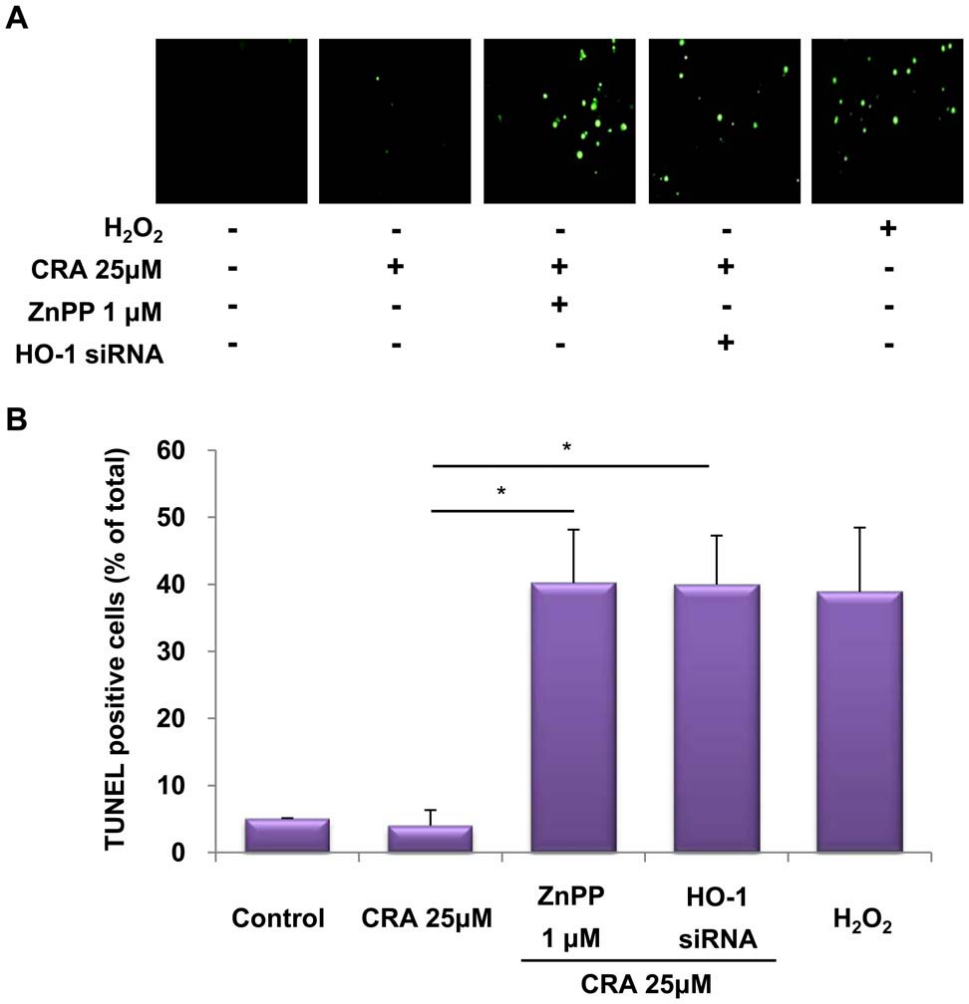
Effect of crotonaldehyde-induced HO-1 inhibition on cell death. Cells were incubated in the absence or presence of ZnPP or HO-1 siRNA for 16 h before the indicated tests were performed. Crotonaldehyde-stimulated HepG2 cells were pretreated for 1 h with 1 µM ZnPP or HO-1 siRNA. Protective effect of HO-1 induction on cell death as determined by *in situ* terminal nick end-labeling (TUNEL). Treatment with H_2_O_2_ (0.5 mM) served as a positive control. Representative images illustrating fluorescent TUNEL (green) staining of cells cultured for 16 h before the indicated tests were performed (A). The graph indicates that inhibition of HO-1 expression in crotonaldehyde-stimulated HepG2 cells show a significant increase in the number of TUNEL-positive cells compared with those of normal and crotonaldehyde-treated cells (B). TUNEL-positive cells were quantified in five random fields in each culture well, and converted in percentages by referring to the total number of cells. Data represent the mean ± SD of three independent experiments. *p<0.005 vs. CRA 25 µM treated cells.

Recent studies suggested that HO-1 exerts a cytoprotective effect of inhibiting apoptosis and maintaining cellular homeostasis [29], [30]. However, the expression of HO-1 is obviously up-regulated in different types of cancer and the elevated expression of HO-1 is reported to be associated with promotion of survival of tumor cells [31], [32]. To further determine whether the increased level of HO-1 activity enhanced by crotonaldehyde confers cytoprotection, HepG2 cells were pretreated with a specific HO-1 inhibitor, ZnPP, or HO-1 siRNA. Crotonaldehyde-stimulated cells were pre-incubated with or without 1 µM ZnPP or HO-1 siRNA and the presence of dead cells was assessed by *in situ* terminal nick end-labeling (TUNEL) staining, which is widely-used in detecting DNA fragmentation *in situ*. This histochemical technique detects the appearance of intensely stained nuclei, which indicates incorporation of labeled dUTP into the 3′-end of fragmented DNA derived from apoptotic nuclei. Crotonaldehyde treatment showed no significant difference in non-treated cells, whereas, inhibition of HO-1 by pre-treatment with ZnPP or HO-1 siRNA resulted in a noteworthy increase in the proportion of TUNEL-positive (apoptotic) cells (Figure 6A and 6B). These results suggested that HO-1 may serve as a key player in crotonaldehyde-stimulated cell survival pathway.

## Discussion

Enhanced protection against apoptosis plays a central role in the cancer-supportive environment and provides significant resistance to the cytotoxicity of chemotherapy [33], [34]. Also, cancer cells tend to develop self-defense mechanisms to survive such adverse conditions. Several studies have demonstrated that Nrf2 signaling is activated in some cancerous tissues, such as lung carcinomas, and the Nrf2 target genes are abnormally up-regulated in some cancer cell lines [35], [36]. As a consequence, this provides cancer cells with a survival advantage against oxidative cytotoxicity. Recently, Nrf2 has been recognized as a double-edged sword; not only does it protect normal cells from oxidants and electrophilic toxicants, but it also induces the survival of cancer cells under anti-cancer therapeutic conditions [37].

Accumulating evidence supports the cytotoxicity of crotonaldehyde, which induces cell death by acute exposure of cells to oxidative stress through consumption of the antioxidant GSH, resulting in marked carbonylation of a wide range of cellular proteins, and triggering carcinogenesis by chronic injury to DNA [38]. Crotonaldehyde is generated in a wide variety of natural and synthetic processes in the human environment. Cigarette smoke is also a major source of crotonaldehyde, which is also endogenously produced during lipid peroxidation [39]. Considering the number of people exposed to crotonaldehyde of ubiquitous exogenous and endogenous sources, investigation of its potential toxicological effects on various diseases is important. In the present study, we focused on the induction of HO-1 expression in crotonaldehyde-stimulated human hepatocellular carcinoma cells via the PKC-δ-p38-Nrf2 signaling pathway and studied its role in adaptive responses in crotonaldehyde-treated cells.

Among the target genes whose expression is up-regulated by Nrf2, HO-1 is the key enzyme that plays a pivotal role in diminishing the oxidative insults, therefore increasing the survival of cells under oxidative stress [40]. The anti-oxidant, anti-apoptotic, and anti-inflammatory effects of HO-1 result in cytoprotective actions in a variety of pathological models [41], [42], [43]. Several reports demonstrated that MAPK, endoplasmic reticulum-localized pancreatic endoplasmic reticulum kinase (PERK), PI3K, and PKC are involved in HO-1 expression and in Nrf2-dependent transcription [23], [24]. PKC-δ and MAPK play key roles in the activation of Nrf2 in association with HO-1 expression [44]. Curcumin induces HO-1 expression via PKC-δ, p38, and Nrf2-ARE pathways in human monocytes [45], whereas the coffee diterpene kahweol induces HO-1 expression via the PI3K and p38/Nrf2 pathway, but not the extracellular signal-regulated kinase (ERK) and c-Jun N-terminal kinase (JNK) pathway [46]. Signaling mechanisms involved in HO-1 induction may depend on cell types and inducers. The present study implicates a role for PKC-δ and p38 in Nrf2-regulated crotonaldehyde-induced HO-1 expression (Figure 3). In a previous study, we also found that PKC-δ and p38 regulate crotonaldehyde-induced HO-1 expression in endothelial cells [47]. However, PKC-δ and p38 inhibitors did not affect crotonaldehyde-induced HO-1 expression in RAW 264.7 macrophages and A549 human lung epithelial cells (unpublished data). In addition, PD98059 and/or LY294002, but not SB203580 or rottlerin, significantly inhibited crotonaldehyde-induced HO-1 expression in macrophages and epithelial cells, suggesting various pathway of activation for crotonaldehyde.

Our results show that the PKC-δ and p38 pathways are required for crotonaldehyde-stimulated expression of HO-1 and induced translocation of Nrf2 in the nucleus. Furthermore, the effect of transient transfection with Nrf2 siRNA on HO-1 induction supports the notion that HO-1 is strongly associated with the Nrf2 transcription factor (Figure 4). The transcription factor Nrf2 plays an essential role in the induction of phase II detoxifying enzyme, including HO-1, consistent with the previous study that Nrf2 can bind to the EpRE/ARE in the promoter regions of many antioxidant and phase II detoxifying genes, such as NADPH quinine oxidoreductase (NQO1), glutamate-cysteine ligase catalytic (GCLC), and HO-1 [48]. Improved expression of the EpRE/ARE-Luc reporter gene by crotonaldehyde suggests that crotonaldehyde can stimulate EpRE/ARE transcriptional activity (Figure 4C). Taken together, these results support the view that crotonaldehyde-stimulated HO-1 induction requires activation of Nrf2- EpRE/ARE in HepG2 cells.

Induction of HO-1 promotes cancer cell growth and survival, enhances cancer cell resistance to apoptotic death, and even stimulates metastasis and angiogenesis [31], [37]. HO-1 could be a survival factor for carcinoma cells by providing an anti-apoptotic effect. We hypothesized that induction of HO-1 by crotonaldehyde would have anti-apoptotic effects that would result in suppression of cell death. In this study, we examined whether the cell-cycle and cell death was influenced by suppression of crotonaldehyde-stimulated HO-1 induction by treatment with the HO-1 specific inhibitor ZnPP or HO-1 siRNA. Inhibition of crotonaldehyde-stimulated HO-1 induction induced accumulation of cells in the sub-G1 phase (Figure 5) and a significant increase of TUNEL-positive (apoptotic) cells (Figure 6). PI staining is a sensitive means of assessing the cell cycle distribution of whole cell populations [49] and the sub-G1 phase of cell cycle content refers to apoptotic cells [50]. We observed that cell cycle arrest and the proportion of TUNEL-positive (apoptotic) cells increased in HO-1 siRNA or ZnPP-treated cells compared to cells treated with crotonaldehyde alone, supporting the view that crotonaldehyde-stimulated HO-1 induction serves as a mechanism for survival cancer cells.

In the present study, we attempted to examine whether crotonaldehyde may induce up-regulation of HO-1 expression through the PKC-δ–p38 MAPK–Nrf2 pathways and whether induction of HO-1 contributes to survival during crotonaldehyde exposure in HepG2 cells. Our results support an anti-apoptotic effect of HO-1 in crotonaldehyde-stimulated HepG2 cells and provide a mechanism by which induction of HO-1 expression by crotonaldehyde may promote tumor resistance.

## Materials and Methods

### Materials

Crotonaldehyde, MTT [3-(4, 5-dimethylthiazol-2-yl)-2, 5-diphenyltetrazoliumbromide] and zinc protoporphyrin (ZnPP) were obtained from Sigma (St. Louis, MO), and TRIzol® was supplied by Invitrogen (Carlsbad, CA). TransPass R2 Transfection Reagent was obtained from New England Biolabs (Hercules, CA). Dulbecco’s modified Eagle’s medium (DMEM), fetal bovine serum (FBS), and tissue culture reagents were obtained from WelGENE Co. (Daegu, Korea). The following antibodies were used; anti-Nrf2 (Santa Cruz Biotechnology, Santa Cruz, CA), anti-Lamin B (Santa Cruz), anti-HO-1 (Epitomics, Burlingame, CA), anti-phospho-p38 (Cell Signaling Technology, Beverly, MA), anti-phospho-PKC-δ (Cell Signaling Technology), anti-p38 (Cell Signaling Technology), anti-PKC-δ (Cell Signaling Technology) and anti-GAPDH (AbFrontier, Seoul, Korea). SB203580 and Rottlerin were purchased from Calbiochem (La Jolla, CA). Nrf2 (SC-37030) siRNA, HO-1 (SC-35554) siRNA and PKC-δ (SC-36253) siRNA were obtained from Santa Cruz and p38 (#6564) siRNA was purchased from Cell Signaling Technology. All other chemicals and reagents were of analytical grade.

### Cell Culture and Viability Measurement

We purchased human hapatocellular carcinoma cells (HepG2) from the ATCC (Rockville, MD). Briefly, cells were cultured in DMEM medium supplemented with 10% heat-inactivated fetal bovine serum, 100 U/ml penicillin, and 100 µg/ml streptomycin at 37°C under 5% CO_2_ and 95% air. For all experiments, the cells were grown to approximately 60–80% confluence.

Cell viability was measured using the conventional MTT assay as described [51].

### Western Blot Analysis

Cells were lysed in lysis buffer containing 1 mM EDTA, 5 µg/ml aprotinin, 2 µg/ml leupeptin, and 1 mM PMSF. Western blot analysis was performed using monoclonal antibody against heme oxygenase-1 (HO-1) and monoclonal antibody against GAPDH. Horseradish peroxidase-conjugated anti-IgG antibodies were used as the secondary antibody to detect the abovementioned protein bands by enhanced chemiluminescence WESTSAVE Up™ (Ab frontier).

### RNA Isolation and Reverse Transcriptase-polymerase Chain Reaction

Total RNA was isolated using TRIzol® and a total of 1 µg of RNA was used for the cDNA synthesis using random primers. Reverse transcription was performed as described previously [52]. PCR was performed using synthesized cDNA as a template and specific primer for HO-1 or GAPDH as a loading control. The primer sequence for mouse HO-1 was 5′-ACATCTATGTGGCCCTGGAG-3′ (forward) and 5′-TGTTGGGGAAGGTGAAGAAG-3′ (reverse). Amplified products were resolved by 1.5% agarose gel electrophoresis, stained with ethidium bromide, and photographed under ultraviolet light.

### Assay for HO Activity

HO enzyme activity was determined, as described previously [53]. The reaction was carried out in the dark for 1 h at 37°C, and the amount of extracted bilirubin was calculated by the difference in absorbance between 464 and 530 nm.

### Nrf2, HO-1, p38, and PKC-δ Silencing by siRNA

Cells were seeded on six-well plates at a density of 2.0 × 10^5^ cells/well and transfected with specific siRNA or scrambled siRNA for 16 h. For each transfection, 1,200 µl of the transfection medium was added to 0.25–1 µg or 10–30 nM of the siRNA duplex/transfection reagent mix (TransPass R2 solution A + B), and the entire volume was added gently to the cells.

### Immunofluorescence Staining

Cells were cultured in a glass culture chamber slide (Falcon Plastics Inc., London Ontario, Canada), and processed for histological and Immunofluorescence analyses. Immunofluorescence method as described previously method [47]. The images were observed under a fluorescence microscope (Eclipse 50i; Nikon, Japan).

### Preparation of Nuclear Proteins

After treatment with crotonaldehyde for 4 h, nuclear extract was prepared, as described previously [52].

### Measurement of Promoter Activity

EpRE/ARE-luciferase (EpRE/ARE-Luc) reporter plasmid was a generous gift from Dr. Park, R. K. (Wonkwang University, Korea). EpRE/ARE -Luc was generated by transfer of the enhancer 2 (E2) and minimal promoter (MP) sequences into the luciferase reporter plasmid pGL3-Basic. Measurement of promoter activity method as described previously method [47].

### Cell Cycle Analysis by Flow Cytometry

Approximately 10^6^–10^7^ cells were harvested by trypsinization and gently pelleted by centrifugation at 200 g for 5 min. After centrifugation, cells were washed in cold PBS and resuspended in PBS. The suspended cells were transferred drop-wise into 4.5 ml of 70% ethanol, and then fixed for more than 2 h. The ethanol-suspended cells were then collected, washed and resuspended in 50 µg/ml propidium iodide (PI, Sigma)/0.1% (v/v) Triton X-100 staining solution with 100 µg/ml RNase A in the dark for 30 min at room temperature. A FACScan (Becton Dickinson, San Jose, CA) was used for analysis of cells. CELLQuest software (Becton Dickinson) was used for data analysis.

### Terminal Deoxynucleotidyl Transferase-mediated dUTP Nick End-labeling (TUNEL) Assay

To measure DNA fragmentation, the commercially available *in* *situ* death detection kit (Roche Diagnostics, Mannheim, Germany) was utilized and measurement of DNA fragmentation method as described previously method [52].

### Statistical Analysis

Statistical significance was estimated by student’s *t*-test and the results were expressed as mean ± S.D.
